# Phosphorus and carbohydrate metabolism contributes to low phosphorus tolerance in cotton

**DOI:** 10.1186/s12870-023-04100-6

**Published:** 2023-02-16

**Authors:** Asif Iqbal, Dong Qiang, Wang Xiangru, Gui Huiping, Zhang Hengheng, Zhang Xiling, Song Meizhen

**Affiliations:** 1 State Key Laboratory of Cotton Biology, Institute of Cotton Research of Chinese Academy of Agricultural Sciences, Zhengzhou Research Base, School of Agricultural Sciences, Zhengzhou University, State Key Laboratory of Cotton Biology, Anyang, Henan 455000 People’s Republic of China; 2 Department of Agriculture, Hazara University, Khyber Pakhtunkhwa 21120 Mansehra, Pakistan; 3 Western Agricultural Research Center of Chinese Academy of Agricultural Sciences, Changji, 831100 Xinjiang China

**Keywords:** Cotton, Low phosphorus tolerance, Root morphology, Carbohydrate accumulation, phosphorus metabolism

## Abstract

Low phosphorus (P) is one of the limiting factors in sustainable cotton production. However, little is known about the performance of contrasting low P tolerant cotton genotypes that might be a possible option to grow in low P condition. In the current study, we characterized the response of two cotton genotypes, Jimian169 a strong low P tolerant, and DES926 a weak low P tolerant genotypes under low and normal P conditions. The results showed that low P greatly inhibited growth, dry matter production, photosynthesis, and enzymatic activities related to antioxidant system and carbohydrate metabolism and the inhibition was more in DES926 as compared to Jimian169. In contrast, low P improved root morphology, carbohydrate accumulation, and P metabolism, especially in Jimian169, whereas the opposite responses were observed for DES926. The strong low P tolerance in Jimian169 is linked with a better root system and enhanced P and carbohydrate metabolism, suggesting that Jimian169 is a model genotype for cotton breeding. Results thus indicate that the Jimian169, compared with DES926, tolerates low P by enhancing carbohydrate metabolism and by inducing the activity of several enzymes related to P metabolism. This apparently causes rapid P turnover and enables the Jimian169 to use P more efficiently. Moreover, the transcript level of the key genes could provide useful information to study the molecular mechanism of low P tolerance in cotton.

**Supplementary Information:**

The online version contains supplementary material available at 10.1186/s12870-023-04100-6.

## Introduction

Phosphorus (P) is one of the major elements required for all physiological processes, reproduction, and environmental adaptation that can maintain sustainable crop production [1]. It is vital for photosynthesis and energy metabolism, biosynthesis of organic compounds, nucleic acids and phospholipids, enzymatic activities, and gene regulation as well as signaling [2]. Generally, the availability of P is low due to precipitation and low mineralization which affect plant growth and productivity. It is documented that about 70% of the world’s cultivated land is P deficient, making P nutrition a high research area of great importance [3]. When P is applied as inorganic fertilizer, it is immobilized due to its high reactivity with calcium and magnesium in alkaline soils and with iron and aluminum in acidic soils [4]. Even under a well-managed P fertilization, plants can only acquire 30% of the applied P, and the rest of P is lost due to fixation and microbial activity. Therefore, P is one of the key problems that restrict plant growth and development in both alkaline and acidic soils [5]. Despite the total amount of P in the soil, the availability of P is low [6] and plants are often facing the P deficiency [5]. As a result, growers applied excess P fertilizers, however, an increase in P fertilization led to an increase in the cost of production and environmental pollution [7]. Therefore, the identification and development of genotypes that can tolerate low P and maintain normal growth under low P is the need of time.

Genotypes of the same species produced different yields under the same nutrient conditions [8]. In terms of P, the variation among contrasting genotypes is the result of many roots and shoots associated mechanisms [8]. But it is difficult to specify the exact mechanism that is responsible for the variation in the yield of contrasting P-efficient genotypes [9], since these mechanisms are different according to crops and nutrient deficiency, and many of them are still to be elucidated [8]. Many researchers have studied one or more mechanisms that explain the high tolerance of P-efficient genotypes under low P condition [6, 10, 11]. It was reported that different species and genotypes of the same species have developed a diverse adaptive mechanism to tolerate low P stress. Among these mechanisms, plants have evolved several morphological, physiological, and biochemical adaptations, such as modification in root system [12], high exudation of organic acids [13] and acid phosphatases [14], and increased the enzymatic activities related to carbohydrate metabolism [15].

Among various responses, modification in the root system is a well-documented response under low P condition [16]. The great response of plants to low P is to stimulate root growth at the expense of shoot growth [17]. Subsequently, some plants proliferate inside the soils to acquire more nutrients by enhancing the root surface area in the soil profile [18]. Moreover, a biochemical adaptation can occur in P-efficient genotypes by producing more root exudates [13] and acid phosphatases which helps to mobilize the bound P and thus increase P uptake [19]. It was observed that transgenic tobacco plants producing more citrate have increased P uptake and utilization efficiency [20]. Therefore, plants that produce and exudes phosphatases can increase P uptake and utilization under low P condition [19]. Low P also reduces leaf photosynthesis but increased the accumulation of starch in plant tissues [21]. Similarly, low P increased soluble sugars, sucrose, and starch contents in beans [22], tomatoes [23], and cotton leaves [24]. Under low P, a significant increase in carbohydrate contents was observed in the Arabidopsis leaves [25], however, an increase in carbohydrate contents inhibits photosynthesis [26], C metabolism, and biomass accumulation [27]. This inhibition is mainly the result of non-stomatal limitations [28], as P is involved in the regulation of RUBP, carboxylation, energy supply, stomatal size, and conductance [29]. Generally, low P reduces crop growth by inhibiting leaf photosynthesis and does not show immediate deficiency symptoms until severe P starvation [30]. Moreover, in low P condition, about 60-90% of root P is remobilized from senescing tissue to the developing seeds [31], however, the P remobilization is low under normal P [30, 32].

Carbohydrates such as sucrose and starch are important for coping with abiotic stresses by improving the osmotic pressure in leaves and roots [33]. Photosynthesis produces carbohydrates in the form of sucrose and starch that are important for cotton growth and development [34] and are the basis for cotton yield and quality [35]. Among them, sucrose is the main form of carbohydrate transport, and it is also a signaling molecule in response to low P [36]. It was reported that carbohydrate in the crop leaves under low P varies among different crops such as sucrose and starch content in beet, rice, maize, and barely leaves increased, while some other studies showed little effect in corn and barely [37]. In the early stage of P deficiency, carbohydrates are transported from the shoot to the root for the development of a better root system, while at the reproductive stage, P deficiency disturbs the synthesis and transportation of carbohydrates, leading to high root-shoot ratios [38]. However, studies have shown that preferential allocation of carbohydrates from shoot to root increases the plant tolerance to low P and is not affecting the growth [39]. Thus, the high root to shoot ratio is not due to the partitioning of carbohydrates but the efficient utilization of carbohydrates by roots under low P condition. Therefore, the carbohydrate metabolism of cotton is not only reflected in the photosynthetic capacity, but also in the temporary storage capacity of the leaves and roots for excess carbohydrate assimilation and the remobilization capacity of the stored carbohydrates when effective photosynthesis is insufficient.

Similarly, low P activates and regulates many enzymes related to the biosynthesis and degradation of carbohydrates (sucrose) such as sucrose phosphate synthase (SPS), fructose 1,6-biphosphatase (FBP), and sucrose synthase (SS) [40, 41]. The combined activities of SPS, FBP, and SS in cotton increased the source-sink sucrose concentration gradient and transportation [42]. Low P affects the enzymatic activities related to carbohydrate metabolism [43] as shown by 76 and 42% reduction in spinach and maize leaves, respectively [44, 45]. The increase in enzymatic activities under low P is inconsistent as SPS and FBP in the sugar beet leaves increased by 97 and 58% [46], while decreased by 4 and 44% in maize leaves, respectively [45]. Meanwhile, low P had a minor effect on the SS activity in the leaves of maize and sugar beet [45, 46]. Similarly, low P had lower SS activity in the shoot and root of tobacco as compared to normal P [47]. Conversely, a higher SS activity was noted in the root tips of beans [48]. Thus, under low P, the enzymatic activities related to carbohydrate metabolism are dependent on species and different tissue of the same species. Moreover, a previous study found carbohydrate accumulation and distribution in cotton leaves under low P [49]. Therefore, carbohydrate assimilation and its metabolic key enzymes in cotton are not only important in the process of assimilates transformation but also play a vital role in the transmission of related stress signals and the control of carbohydrate assimilation processes. The carbohydrate contents and related enzymatic activities in cotton were closely related in response to low P stress.

Cotton is the leading fiber crop grown throughout the world, providing raw materials to the textile industry [50, 51]. China is one of the leading cotton producers, consumers, and importers in the world [52, 53]. In China, most of the cotton is shifted from the Yellow River and Yangtze River valley to Xinjiang province [54]. However, Xinjiang is an arid region having low precipitation and high surface evaporation and are therefore facing the issues like scarcity of water resources [55] and low nutrient availability, especially P [56]. Studies have found that the availability of P is very poor in Xinjiang soil due to its calcareous nature which leads to poor plant growth, dark green leaves, yellowing of leaves, flower bud necrosis, and finally poor cotton yield and quality [57]. Therefore, the use of chemical fertilizer is increased in the last few decades and it might increase by 2% in the future to maintain the current yield [58]. Moreover, the increase in P fertilization will increase the cost of production and environmental pollution [59]. However, without the addition of P fertilizer over a long period, the soil available P concentration will gradually decrease and the crop yield and quality will decrease accordingly (Yao et al., 2012). Thus, due to insufficient soil available P and rock phosphate resources in China, it is an important task for cotton researchers to develop low P tolerant cotton genotypes that can produce high yield and fiber quality under relatively low P. Previously, we have identified two cotton genotypes Jimian169 and DES926 as strong tolerance to low P and weak tolerance to low P genotypes, respectively. However, the morphological, physiological, biochemical, and molecular mechanisms of low P tolerance is still to be elucidated. Therefore, it is of main importance to study the hypothesis that cotton genotype Jimian169 has a different mechanism of low P tolerance than DES926. This study will be useful to understand the mechanism of low P tolerance and provide basis for the genetic development of low P tolerant cotton genotype.

## Materials and methods

### Plant materials

Based on the multi-year P fertilizer experiments in the experimental farm of the Cotton Research Institute of the Chinese Academy of Agricultural Sciences (CRI, CAAS), we have found that the amount of available P (3 ± 0.5) mg kg^− 1^) in the soil is low and the cotton genotypes respond differently during the cotton growth period. Therefore, the current study is very important to understand the genotypic difference under low and normal P conditions. Moreover, the selection of the strong and weak low P tolerant cotton genotypes is the basis for the current study. Previously, we studied the agronomic performance and P use efficiency (PUE) of 384 cotton genotypes under low (LP; 0.01 mM KH_2_PO_4_) and normal (NP; 1 mM KH_2_PO_4_) P conditions, and based on dry biomass and PUE, 30 cotton genotypes were selected. The selected 30 cotton genotypes were again grown under low and normal P conditions in pot and hydroponic culture and finally two cotton genotypes (Jimian169; strong low P tolerant and DES926; weak low P tolerant) with contrasting low P tolerance were identified.

### Experimental design

A greenhouse hydroponic experiment was conducted at CRI, CAAS, Anyang, China. According to the previous study, two cotton genotypes Jimian169 (strong tolerance to low P) and DES926 (weak tolerance to low P) were used in the experiment. The genotypes selected from the previous experiments were used in this study. The seeds of selected contrasting low P tolerant cotton genotypes were kindly provided by the ICR, CAAS, China. The selected seed permission was granted from the respective authority. Healthy seeds of both cotton genotypes were sown in sterilized sand in an incubator for 1 week. After germination, uniform healthy plants were selected and transplanted in a plastic container (7 L) in a growth condition of 16/8 h light/dark cycle, 28 °C temperature, and 60% relative humidity. Half concentration Hoagland solution was applied during the first week followed by full strength as mentioned in our previous study [60]. Further, seedlings with two true leaves were exposed to low (0.01 mM KH_2_PO_4_) and normal (1 mM KH_2_PO_4_) P conditions. The seedlings were aerated with an electric pump and the solutions were renewed once a week. After obvious morphological variation, the 4 week old seedlings were harvested and various morphophysiological traits were measured.

At the same time, a pot experiment was conducted in the greenhouse at the CRI, CAAS, Anyang, Henan province, China. The soil for the pot experiment was collected from 0 to 20 cm low fertile arable soil from the cotton field. The two contrasting low P tolerant cotton genotypes Jimian169 and DES926 were sown in the plastic bucket (diameter: 12 cm, height: 10 cm) having soil collected from the cotton field under low and normal P conditions. The other nutrients were used at the recommended levels. All the management practices were kept the same for all the buckets except P levels.

### Plant morphology

From each treatment, six plants were randomly selected and the shoot length was measured with the help of calibrated scale [61]. After harvesting, the plants were divided into roots and shoots and subsequently dried at 105 °C for 1 h followed by 80 °C for 48 h. After complete drying, the shoot, root, and total dry matter were determined using an electric balance. At the same time, the roots of half of the plants from each genotype were scanned and analyzed through WinRHIZO root analyzer system [62] to determine the root length, root surface area, and root volume. Root length ratio (root length/whole plant dry weight), root mass ratio (root dry weight/whole plant dry weight), root thickness (root length/root volume), and root density (root dry weight/root volume) were measured as mentioned in our previous study (A. Iqbal, Dong, et al., 2020).

### Measurements of photosynthetic and chlorophyll traits

The photosynthetic traits were measured from the third fully expanded leaf by using the photosynthetic machine (Li-Cor 6800, USA) from 9:00 to 11:00 a.m. [63]. Carbon dioxide concentration inside the chamber was maintained at 400 ± 1 μmoL CO_2_ (mol air)^− 1^, and the light intensity was set as 1000 μmol photon m^− 2^ s^− 1^. About 50 mg of fresh leaf sample was used to measure chlorophyll and carotenoid contents. The collected samples were cut into small pieces and incubated overnight in acetone: ethanol (1:1) solution for 48 h at 25 °C. The absorbance values for chlorophyll and carotenoid contents were measured according to our previous study [64].

### Determination of phosphorus concentration and use efficiency

P concentration in root and shoot tissues were measured according to Kjeldahl method [65]. The grounded sample of 0.2 g from each tissue was digested with H_2_SO_4_-H_2_O_2_, and the final P concentration was analyzed using the Bran + Luebbe Continuous-Flow Auto Analyzer III. The various PUE related definitions were measured according to our previous study [60].

(1) Total P accumulation (TPA) calculated as the P concentration x total plant dry matter;

(2) P utilization efficiency (PUtE) calculated as the total plant dry matter divided by P concentration;

(3) P uptake efficiency (PUpE) calculated as TPA divided by root dry matter.2.6 Determination of carbohydrate contents and enzymatic activities related to phosphorus and carbohydrate metabolism.

The carbohydrates contents like glucose, fructose, sucrose, and starch were measured according to the instructions provided by the company. A fresh sample of 0.1 g from root and shoot was added in a 5 ml 80% ethanol solution. The extract was centrifuged at 8000×g for 10 minutes and the supernatant collected was stored at 4 °C for the next analysis. Further, the supernatant was incubated for 48 h at 37 °C in acetate buffer (4.5 mmol·L^− 1^) and α-glucoamylase (0.5%, w/v), and water. The final values of glucose, fructose, sucrose, and starch were recorded at 505 nm, 480 nm, 480 nm, and 620 nm, respectively. The glucose content was expressed as μmol g^− 1^ FW, whereas fructose, sucrose, and starch as mg g^− 1^ FW.

The activities of acid phosphatase (ACP), alkaline phosphatase (ALP), and phosphofructokinase (PFK) were measured according to the protocols provided by the company (Suzhou Comin Biotechnology, Suzhou, China). The root and shoot fresh weight was homogenized with sodium acetate buffer (0.1 M) and centrifuged at 8000 g at 4 °C. The extracts were incubated under dark condition for 30 minutes at room temperature. The absorbance of ACP, ALP, and PFK was measured at 405 nm, 510 nm, and 340 nm. The activities of APA, ALP, and PFK were expressed as μmol min^− 1^ g^− 1^.

The sucrose synthase (SS) and sucrose phosphate synthase (SPS) activities were determined by using the commercially available kits provided by the manufacturer. The fresh samples of about 0.1 g from root and shoot were homogenized in 1 ml extraction buffer and were centrifuged. The enzyme reaction was measured according to the protocol and the final values were recorded at 480 nm using a spectrophotometer. The activities of SS and SPS were expressed as μg min^− 1^ g^− 1^ FW.

Phosphoenolpyruvate carboxylase (PEPC) was extracted and measured according to the commercial chemical kits in accordance with the manufacturer. About 0.1 g fresh root and shoot samples were halogenated in 1 ml buffer (50 mM Hepes-KOH), 1 mM EDTA, 1 mM EGTA, 10% glycerol, 1 mM DTT, 12 mM MgCl_2_, 2 mM benzamidine and 2 mM e-aminon-caproic acid. Further, the solution was kept at 80 °C until the analysis of PEPC. The reaction was started by adding 3.25 mmol phosphoenolpyruvate at 30 °C. The absorbance was measured at 340 nm and expressed as nmol min^− 1^ g^− 1^ FW.

Fructose 1,6-biphosphatase (FBP) was determined using the commercially available kits from Suzhou Comin Biotechnology, China. The root and shoot samples (0.1 g) were mixed in 50 mM Hepes-HCl (pH 7.6), 5 mM MgCl_2_, 10 mM β-mercaptoethanol, 0.25 mM fructose 1,6-biphosphate, 10 mM KF, and 100 μL of extract. After that, the solution was kept in dark for 10 minutes at 25 °C followed by adding the trichloroacetic acid (30%). The Pi released was measured according to the instructions provided by the company and the final values were obtained at 340 nm. FBP activity was expressed as nmol min^− 1^ g^− 1^ FW.

### Determination of malonaldehyde contents and antioxidant enzymatic activities

The malonaldehyde (MDA) content in root and shoot was measured according to the standard protocol [66].

For the determination of antioxidant enzymes activities, 0.2 g fresh samples was obtained by removing the midrib portion. The sample was washed, dried, powdered, and homogenized in 5 mL chilled sodium phosphate buffer (50 mM, pH 7.8). The sample was centrifuged at 12,000×g for 20 min at 4 °C. The supernatant was used to measure superoxide dismutase, peroxidase, and catalase enzymes activities, and results were expressed as U mg^− 1^ min^− 1^ FW [64, 67].

Superoxide dismutase (SOD) activity was determined by measuring the photoreduction of nitroblue tetrazolium (NBT) at 560 nm. About 20 μL of enzyme extract was added to the reaction mixture of 0.3 mL methionine (13 mM), 1.5 mL phosphate buffer (50 mM, pH 7.8), 0.3 mL EDTA-Na_2_ (0.1 mM), 0.3 mL NBT (750 mM), 0.3 mL riboflavin (20 M), and 0.3 mL distilled water. The reaction mixture tubes were put in 15 W lamps light for 10 min and then transferred to dark for 15 min, and absorbance was recorded at 560 nm using the UV spectrophotometer.

Peroxidase (POD) activity was determined at 25 °C guaiacol. In the presence of H_2_O_2_, POD catalyzes the conversion of guaiacol to tetra-guaiacol. Amounts of 0.1 mL H_2_O_2_ (300 mM), 2.7 mL potassium phosphate buffer (25 mM, pH 7.0), 0.1 mL guaiacol (1.5% v/v), 2 mM EDTA solution, and 0.1 mL enzyme extract made up the reaction mixture. A spectrophotometer was used to measure the absorbance at 470 nm every 30 s for up to 2 min.

The activity of the catalase (CAT) enzyme was determined using a previously described method that involves calculating the reduction in H_2_O_2_ absorption at 240 nm. The reaction buffer contained 15 mM hydrogen peroxide (H_2_O_2_) and 50 mM potassium phosphate buffer at a pH of 7.0. Next, 100 μL of enzyme extract was added to the reaction mixture for the reaction initiation. The extinction coefficient of 40 mM^− 1^ cm^− 1^ was used to determine the quantity of H_2_O_2_ in the reaction mixture after 1 min, indicating the activity of CAT.

### Determination of gene transcripts involved in phosphorus and carbohydrate metabolism

Based on the transcriptomic data (data not published), essential members of P metabolism (PAP1, PFK-ALPHA, PFK-BETA, and PHT2) and carbohydrate metabolism (FBP, PEPC16, SPS1, and SS) were selected for quantitative real-time PCR (qRT-PCR) analysis. The samples collected at the end of the hydroponic experiment from both cotton genotypes under low and normal P conditions were used for qRT-PCR. Total RNA from each sample was extracted using the Trizol method. About 0.5 μg of total RNA was reverse transcribed into single-stranded cDNA using the PrimeScript RT Master Mix, and then the genomic DNA was digested with DNA remover as suggested in the protocol. The gene-specific primers and the housekeeping histone3.3 (ATG09810) were designed using primer-blast (http:/www.ncbi.nlm.nih), and are provided in Supplementary Table S1. The qRT-PCR was performed in 20 μl reaction mixture containing 2 μl cDNA, and 10 μl LightCycler480 SYBERGREEN1 Master MIX (TaKaRa) on an ABI7500 system. The PCR reaction consisted of preincubation at 95 °C for 30 s, then 40 cycles of 95 °C for 5 s, and 60 °C for 34 s. Finally, the expression levels were calculated using the 2^-ΔCt^.

### Statistical analysis

The data were arranged in excel and analyzed by two-way ANOVA in a split-plot arrangement using Statistix 10 software. The P conditions were considered as the main plot, whereas cotton genotypes were used as a subplot factor. The means were separated by the least significance difference test at a 5% level of significance. Principal component analysis and correlation analysis were performed in OriginPro (2018) (b9.2.214, OriginLab Corporation, Northampton, MA, USA). All the figures expressed as mean ± standard error were drawn in Graphpad Prism 8.

## Results

### Genotypic variation in plant morphology and leaf physiology

**Fig. 1:**
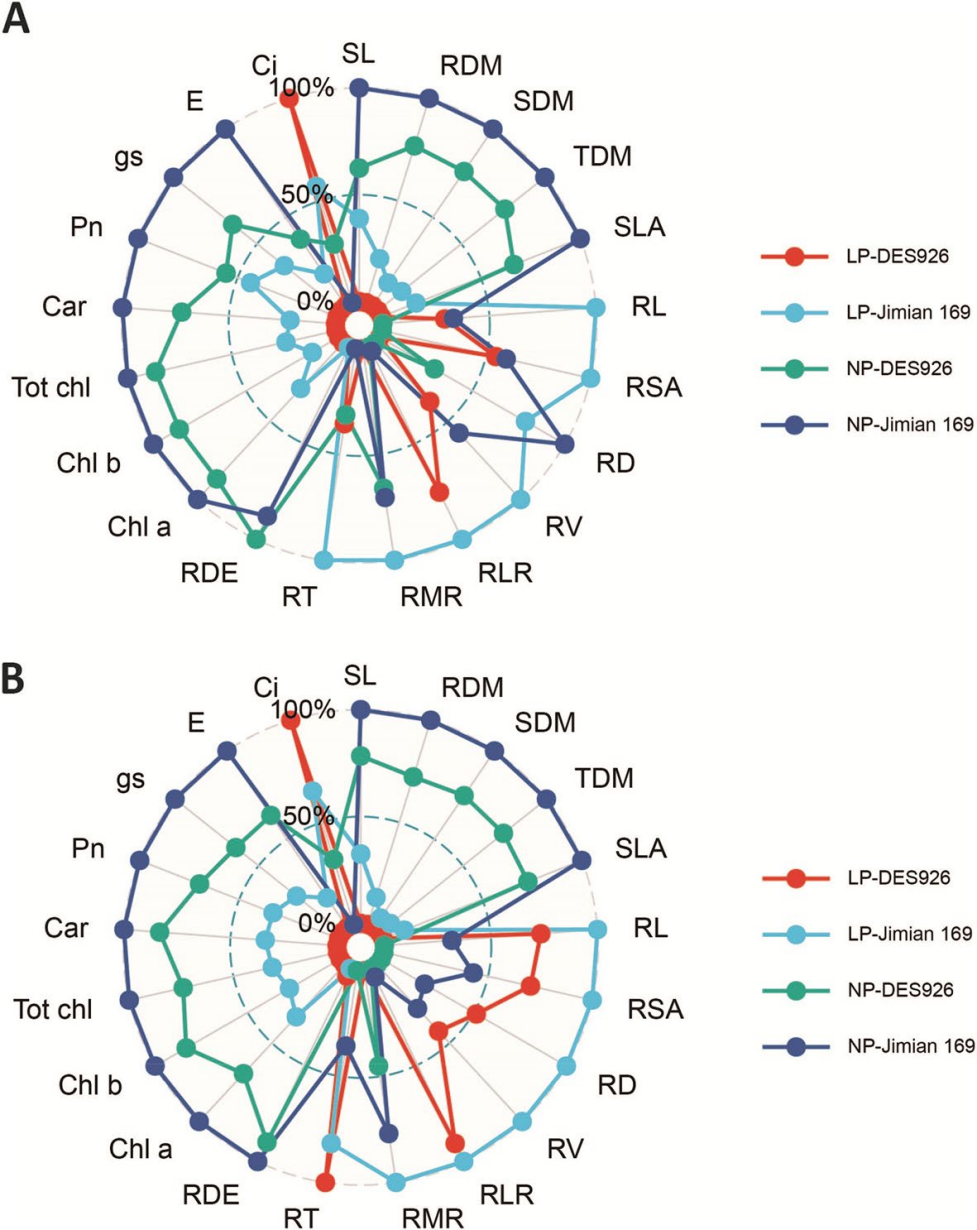
Radar plot represents the genotypic variations in different morphological and physiological traits in hydroponic (**A**) and pot (**B**) cultures under low (LP) and normal (NP) P conditions

In comparison with Jimian169, the shoot length, root dry matter, shoot dry matter, total plant dry matter, and single leaf area of DES926 decreased under low P by 32.7, 15.9, 9.2, 10.3, and 37.5% in hydroponic culture, while 22.6, 10.6, 4.1, 5.2, and 20.9% in pot culture, respectively (Fig. 1). However, under normal P, a reduction of 20.8 and 10.3% for shoot length, 10.9 and 13.1% for root dry matter, 10.5 and 11.2% for shoot dry matter, 10.6 and 11.5% for total dry matter, 25.8 and 19.2% for single leaf area was measured for DES926 in hydroponic and pot cultures (Fig. 1).

Except for root thickness and density, decreases in traits contributing to root development were observed under low P, however, the reduction was more DES926 as compared to Jimian169. Among them, in hydroponic and pot cultures, respectively, a reduction of 21.7 and 10.3% for root length, 11.0 and 4.9% for root surface area, 25.5 and 13.6% for root diameter, 15.5 and 14.1% for root volume, 12.7 and 5.6% for root length ratio, 6.4 and 5.7% for root mass ratio was measured under low P condition (Fig. 1). Similarly, root length (12.7 and 16.7%), root surface area (16.0 and 7.9%), root diameter (21.3 and 8.3%), root volume (16.0 and 8.3%), root length ratio (2.4 and 6.0%), and root mass ratio (0.3 and 1.8%) of DES926 decreased under normal P as compared to Jimian169 (Fig. 1).

Most of the leaf physiological traits were reduced under low P in both hydroponic and pot cultures. However, the decreases were much higher in DES926 than Jimian169, as shown by photosynthesis reduced by 17.6 and 11.8%, stomatal conductance with a reduction of 6.6 and 7.7%%, transpiration rate decreased by 3.8 and 3.4%%, chlorophyll a declined by 12.0 and 21.1%, chlorophyll b reduced by 7.9 and 19.8%, total chlorophyll with a reduction of 10.9 and 20.7%, and carotenoid contents decreased by 10.3 and 20.2% in hydroponic and pot cultures, respectively (Fig. 1). However, under normal P condition, the reduction in DES926 as compared to Jimian169 was 14.6 and 8.7% for photosynthesis, 6.1 and 8.5% for stomatal conductance, 14.6 and 6.3% for transpiration rate, 4.2 and 13.5% for chlorophyll a, 5.3 and 8.0% for chlorophyll b, 4.5 and 11.8% for total chlorophyll, 9.8 and 7.2% for carotenoid contents were measured in hydroponic and pot cultures, respectively (Fig. 1).

### Genotypic variation in PUE traits

**Fig. 2:**
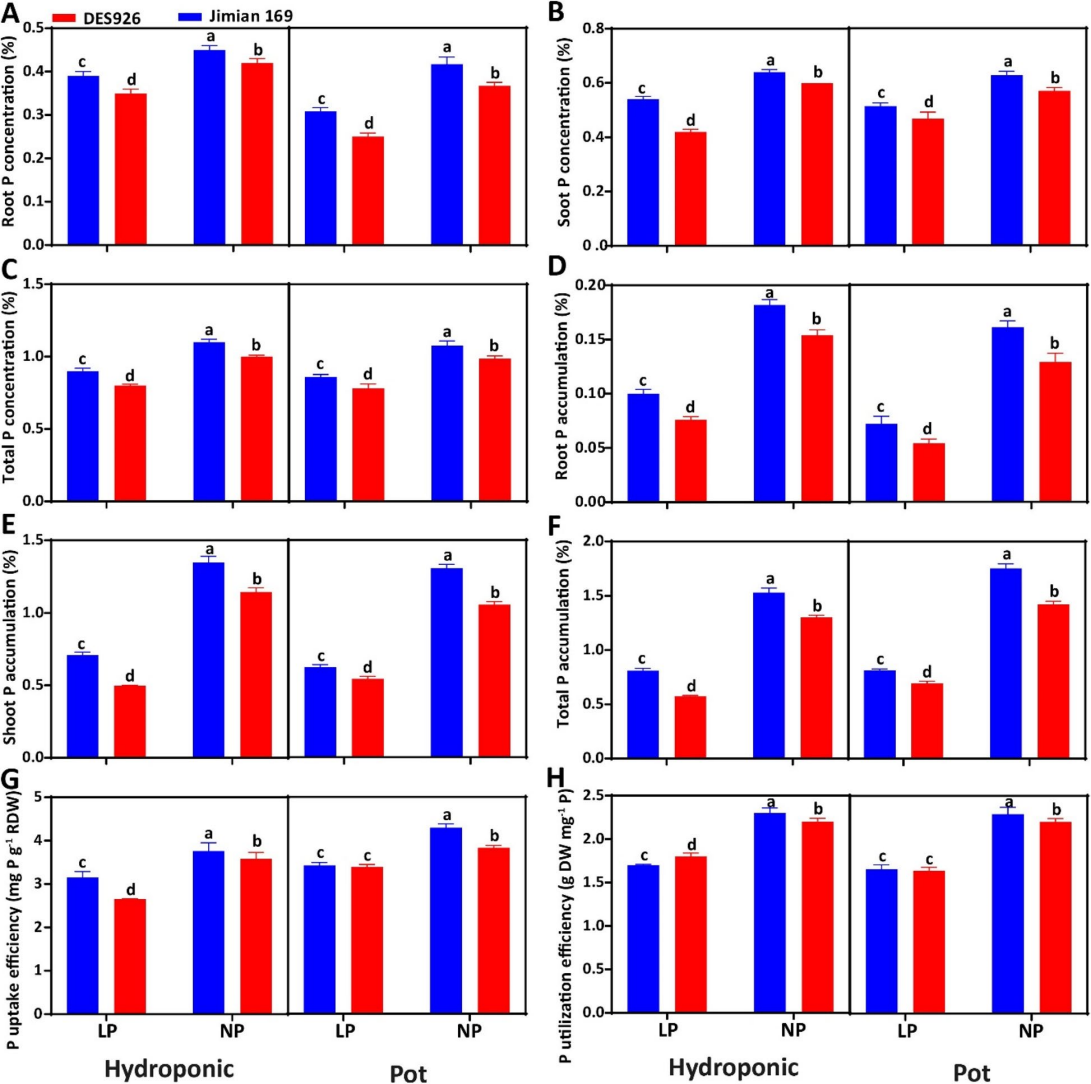
**A** Root P concentration (%), **B** shoot P concentration (%), **C** total P concentration (%), **D** root P accumulation (%), **E** shoot P accumulation (%), **F** total P accumulation (%), **G** P uptake efficiency (mg P g^− 1^ RDW), and **H** P utilization efficiency (g DW mg^− 1^ P) of Jimian169 and DES926 under low and normal P conditions in hydroponic and pot cultures

The root, shoot, and total P concentration abruptly dropped under low P with an approximate reduction of 15.1, 22.5, and 19.4% in hydroponic culture, while 21.0, 16.4, and 18.4% in pot culture, respectively (Fig. 2A-C). In comparison with DES926, the root, shoot, and total P concentration of Jimian169 increased in both hydroponic (6.8, 13.2, and 10.6%) and pot cultures (7.8, 7.3, and 7.5%). Compared to normal P, a significant reduction in the root (46.6 and 52.8%), shoot (51.5 and 58.8%), and total P accumulation (51.1 and 52.2%) were observed under low P. Between the genotypes, root, shoot, and total P accumulation was increased in Jimian169 by 18.2, 20.2, 19.9% in hydroponic culture, while 19.4, 16.8, and 17.3% in pot culture, respectively (Fig. 2D-F). Similarly, low P reduced PUpE (21.0 and 16.6%) and PUtE (22.5 and 25.2%) than normal P in both hydroponic and pot cultures. In comparison with DES926, PUpE and PUtE of Jimian169 increased by 9.8 and 2.1% in hydroponic and 5.2 and 2.6% in pot culture, respectively (Fig. 2G-H).

### Genotypic variation in enzymes related to phosphorus metabolism

**Fig. 3:**
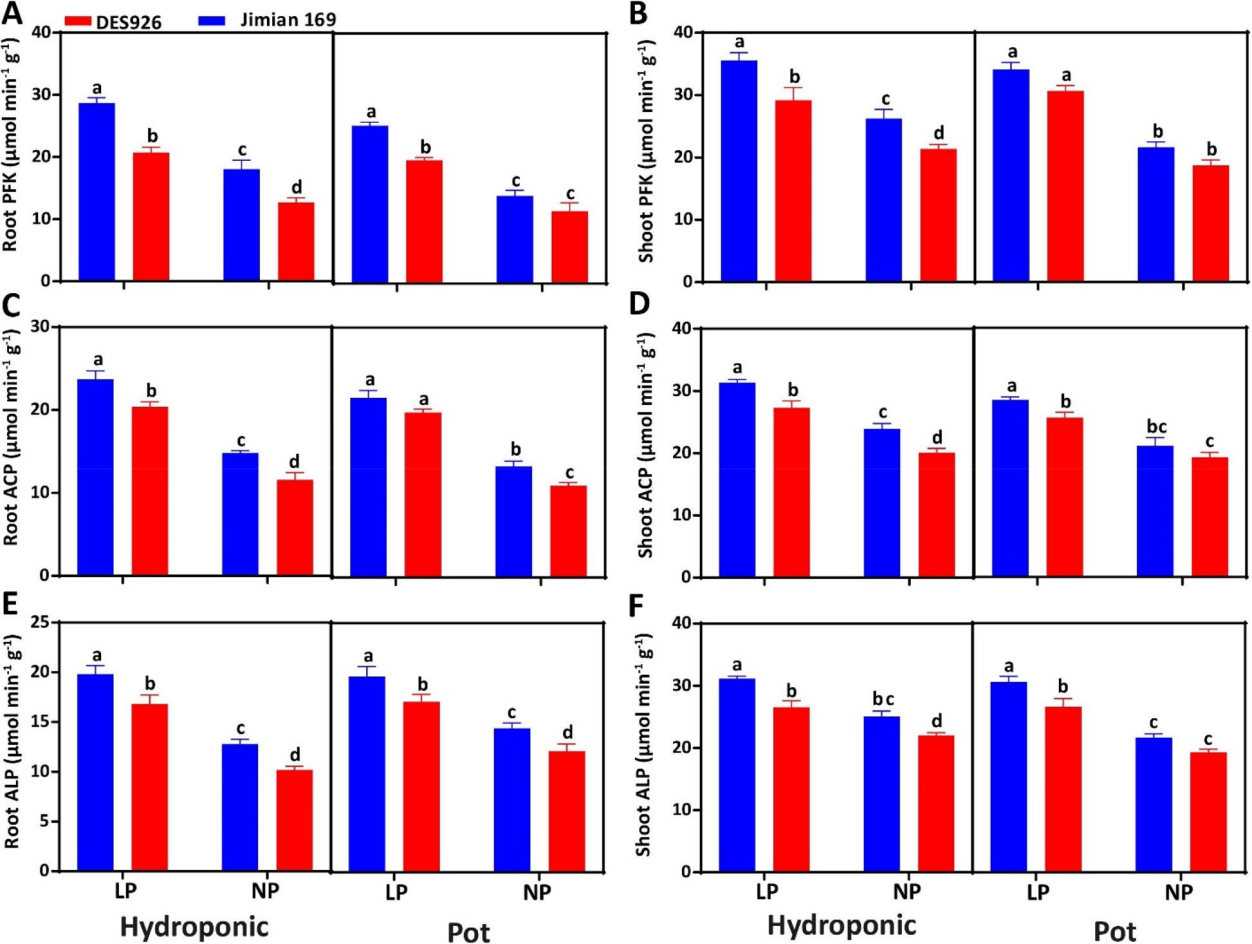
**A** Root phosphofructokinase activity (PFK; μmol min^− 1^ g^− 1^), **B** shoot phosphofructokinase activity (PFK; μmol min^− 1^ g^− 1^), **C** root acid phosphatase activity (ACP; μmol min^− 1^ g^− 1^), **D** shoot acid phosphatase activity (ACP; μmol min^− 1^ g^− 1^), **E** root alkali phosphatase activity (ALP; μmol min^− 1^ g^− 1^), **F** shoot alkali phosphatase activity (ALP; μmol min^− 1^ g^− 1^) of Jimian169 and DES926 under low and normal P conditions in hydroponic and pot cultures

The results showed that low P enhanced the activities of root PFK, shoot PFK, root ACP, shoot ACP, root ALP, and shoot ALP activities by 60.6, 36.0, 67.0, 33.3, 59.5, 22.5% in hydroponic culture, while 75.8, 60.6, 69.9, 34.1, 38.3, and 39.6% in pot culture, respectively (Fig. 3). Moreover, the activities of root PFK (28.4 and 20.3%), shoot PFK (18.2 and 11.4%), root ACP (17.0 and 11.7%), shoot ACP (33.3 and 9.5%), root ALP (59.5 and 38.3%), and shoot ALP (22.5 and 39.6%) were higher in Jimian169 as compared to DES926 (Fig. 3).

### Genotypic variation in related to carbohydrates metabolism

**Fig. 4:**
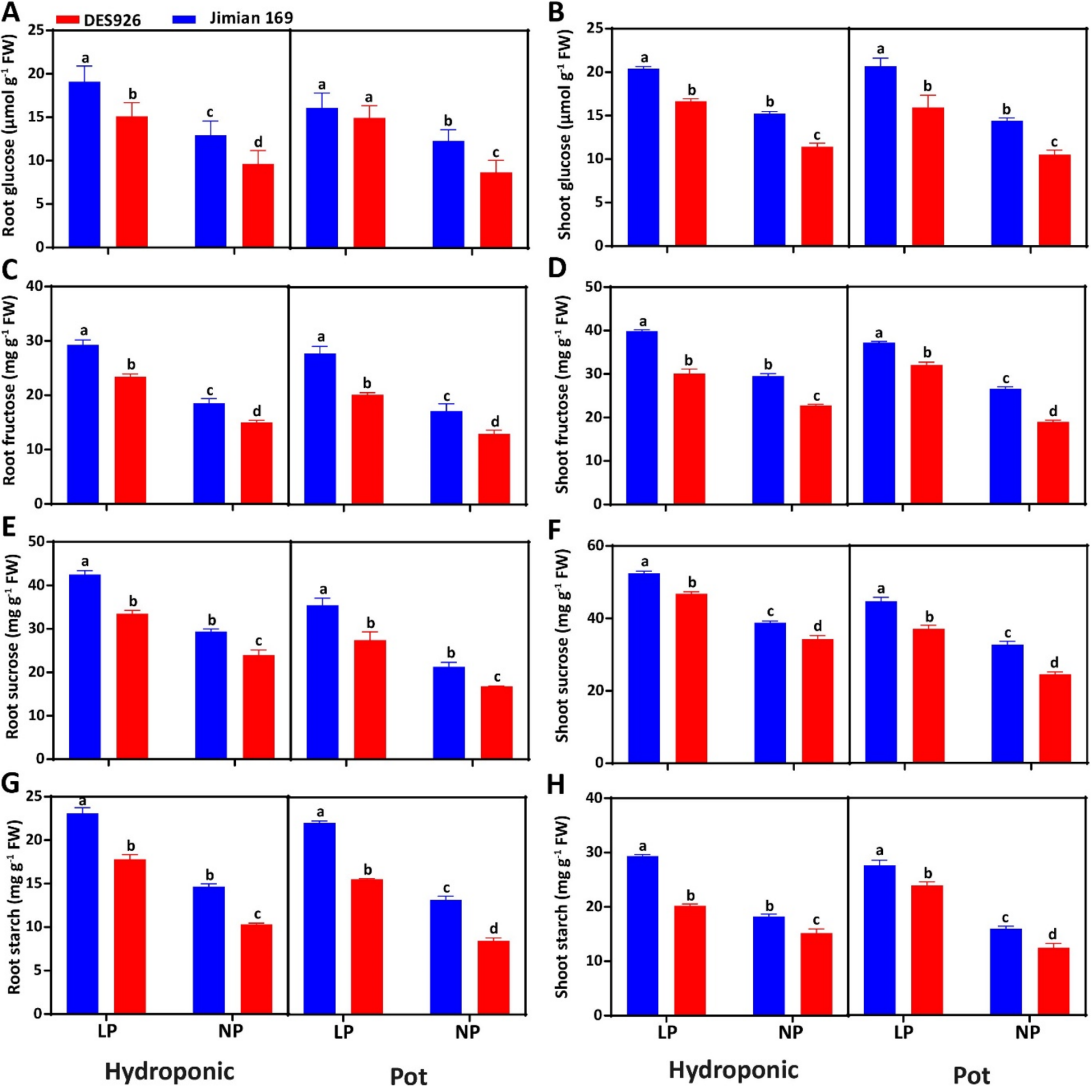
**A** Root glucose content (μmol g^− 1^ FW), **B** shoot glucose content (μmol g^− 1^ FW), **C** root fructose content (mg g^− 1^ FW), **D** shoot fructose content (mg g^− 1^ FW), **E** root sucrose content (mg g^− 1^ FW), **F** shoot sucrose content (mg g^− 1^ FW), **G** root starch content (mg g^− 1^ FW), and shoot starch content (mg g^− 1^ FW) of Jimian169 and DES926 under low and normal P conditions in hydroponic and pot cultures

The root SS (32.9 and 30.9%), shoot SS (32.2 and 39.1%), root SPS (33.5 and 42.4%), and shoot SPS (22.5 and 24.2%) activities were greatly inhibited by low P than normal P (Fig. 4). In comparison with DES926, the root SS, shoot SS, root SPS, and shoot SPS in Jimian169 increased by 17.4, 21.7, 20.0, and 12.6% in hydroponic and by 18.4, 22.7, 22.4, and 15.5% in pot culture, respectively. Similarly, low P reduced root PEPC, shoot PEPC, root FBP, and shoot FBP by 50.7, 35.9, 59.4, and 46.9% in hydroponic and by 61.1, 45.3, 53.1, and 60.3% in pot cultures as compared to normal P, respectively. Compared to DES926, the genotype Jimian169 had significantly higher root PEPC (22.9, and 26.7%), shoot PEPC (21.1 and 32.3%), root FBP (29.4 and 10.4%), and shoot FBP (25.4 and 20.5%) in both hydroponic and pot cultures (Fig. 4).

### Genotypic variation in tissue carbohydrate contents

**Fig. 5:**
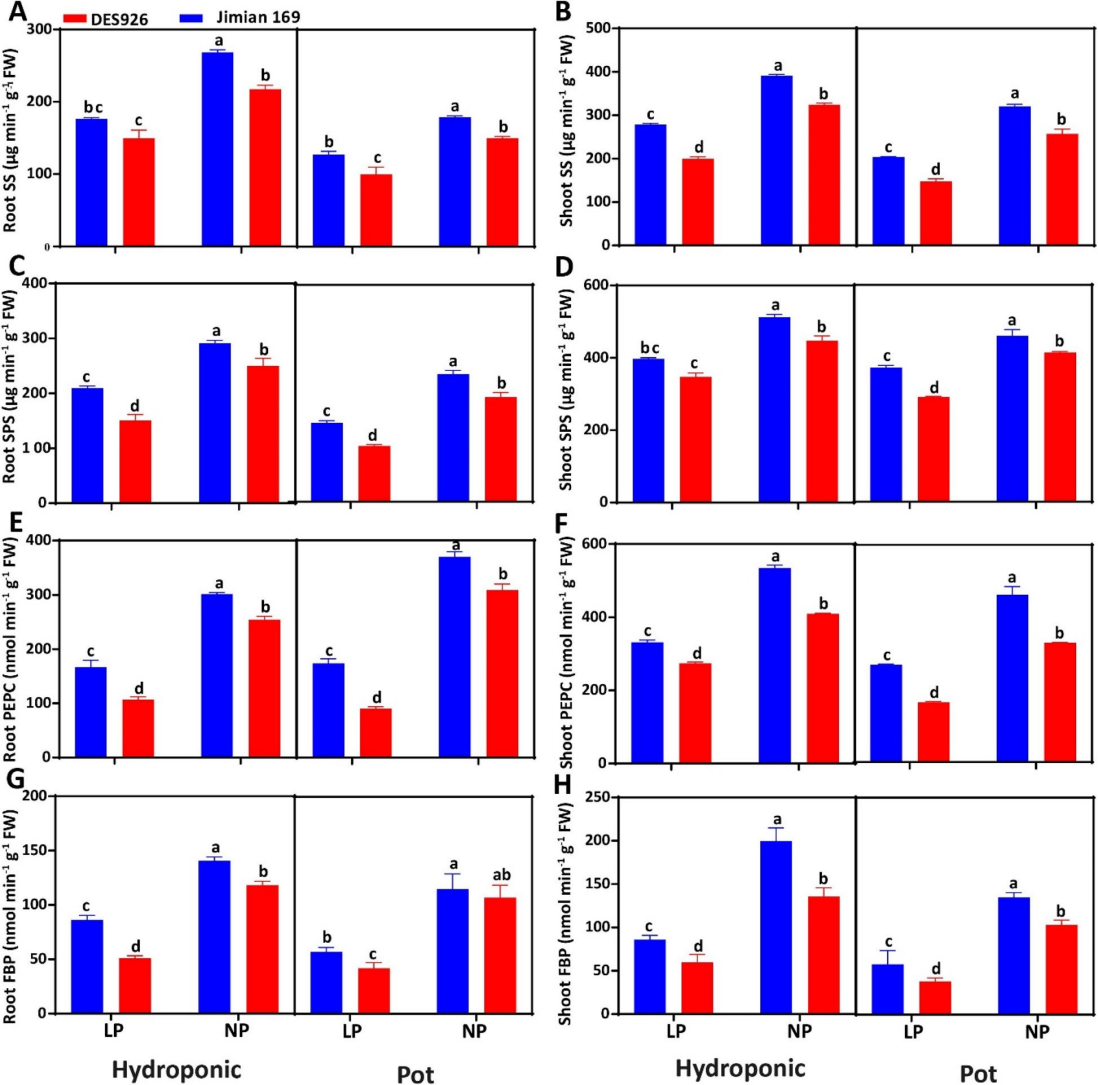
**A** Root sucrose synthase activity (SS; μg min^− 1^ g^− 1^ FW), **B** shoot sucrose synthase activity (SS; μg min^− 1^ g^− 1^ FW), **C** root sucrose phosphate synthase activity (SPS; μg min^− 1^ g^− 1^ FW), **D** shoot sucrose phosphate synthase activity (SPS; μg min^− 1^ g^− 1^ FW), **E** root phosphoenolpyruvate carboxylase activity (PEPC; nmol min^− 1^ g^− 1^ FW), **F** shoot phosphoenolpyruvate carboxylase activity (PEPC; nmol min^− 1^ g^− 1^ FW), **G** root fructose 1,6-bisphosphatase activity (FBP; nmol min^− 1^ g^− 1^ FW), and **H** shoot fructose 1,6-bisphosphatase activity (FBP; nmol min^− 1^ g^− 1^ FW) of Jimian169 and DES926 under low and normal P conditions in hydroponic and pot cultures

Compared to normal P, low P significantly increased root glucose, shoot glucose, root fructose, shoot fructose, root sucrose, shoot sucrose, root starch, and shoot starch increased by 51.7, 38.9, 57.1, 25.3, 42.4, 35.8, 63.7, and 48.4% in hydroponic culture and by 32.4, 31.9, 37.1, 34.3, 39.4, 30.0, 42.4, and 45.0% in pot culture, respectively (Fig. 5). Irrespective of the P condition, the root glucose (22.7 and 16.8%), shoot glucose (21.1 and 24.6%), root fructose (19.7 and 26.3%), shoot fructose (23.8 and 20.1%), root sucrose (20.1 and 22.1%), shoot sucrose (11.2 and 20.5%), root starch (25.5 and 31.8%), and shoot starch (25.6 and 16.6%) contents were higher in Jimian169 than DES926 (Fig. 5).

### Genotypic variation in malondialdehyde content and antioxidant enzymes

**Fig. 6:**
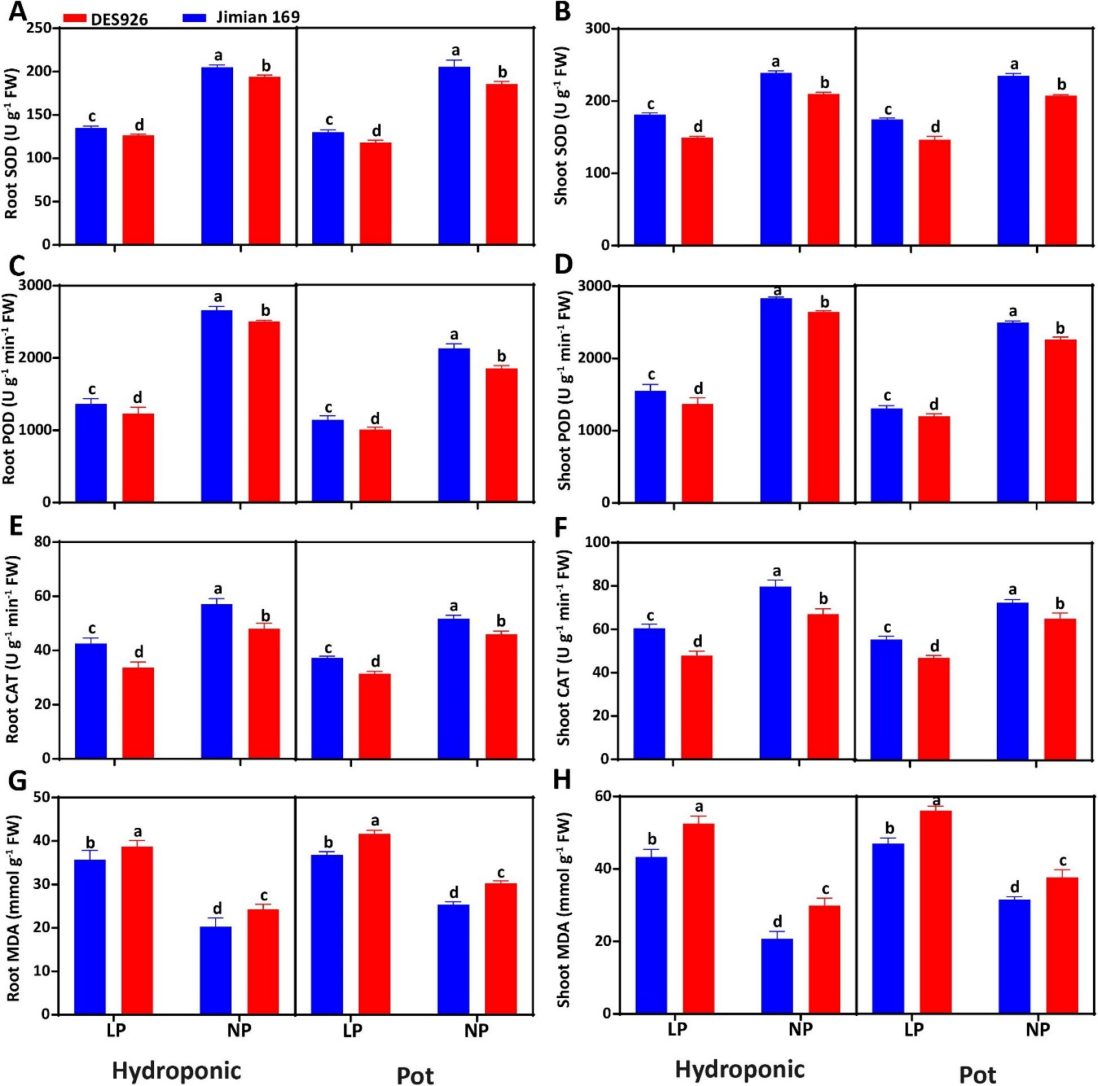
**A** Root superoxide dismutase activity (U g^− 1^ FW), **B** shoot superoxide dismutase activity (U g^− 1^ FW), (**C**) root peroxidase activity (POD; U g^− 1^ min^− 1^ FW), **D** shoot peroxidase activity (POD; U g^− 1^ min^− 1^ FW), **E** root catalase activity (CAT; U g^− 1^ min^− 1^ FW) **F** shoot catalase activity (CAT; U g^− 1^ min^− 1^ FW), **G** root malondialdehyde content (MDA; mmol g^− 1^ FW), and **H** shoot malondialdehyde content (MDA; mmol g^− 1^ FW) of Jimian169 and DES926 under low and normal P conditions in hydroponic and pot cultures

The results showed that the activities of root SOD (34.4 and 35.9%), shoot SOD (26.3 and 27.5%), root POD (49.7 and 44.7%), shoot POD (46.7 and 47.4%), root CAT (27.4 and 29.6%), and shoot CAT (26.1 and 25.4%) were greatly inhibited under low P as compared to normal P in both hydroponic and pot cultures (Fig. 6). In comparison with DES926, the activities of root SOD, shoot SOD, root POD, shoot POD, root CAT, and shoot CAT in Jimian169 increased by 5.7, 14.5, 7.2, 8.5, 18.0, and 18.1% in hydroponic and by 8.8, 13.6, 10.9, 9.0, 13.1, and 12.3% in pot culture, respectively. Malondialdehyde (MDA) content was greatly induced by low P in the roots (67.0 and 41.0%) and shoot (89.5 and 49.0%) of both cotton genotypes in hydroponic and pot cultures. The MDA content was significantly higher in the roots (12.7 and 15.8%) and shoots (28.7 and 19.4%) of DES926 as compared to Jimian169 in both hydroponic and pot cultures, respectively (Fig. 6).

### Genotypic variation in the transcript levels of key genes involved in carbohydrate and phosphorus metabolism

**Fig. 7:**
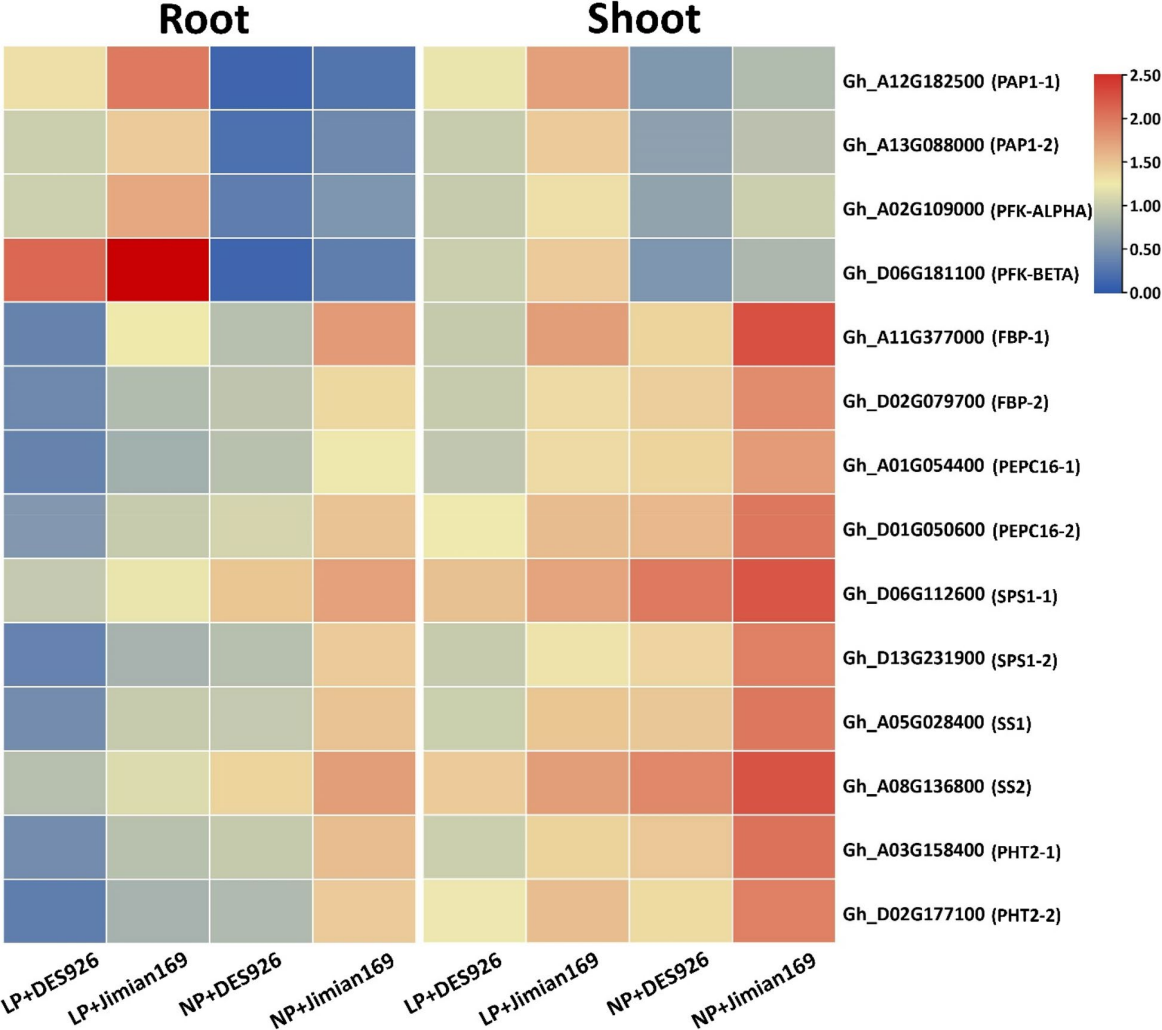
The expression analysis of the key genes involved in the P metabolism (PAP1, PFK-ALPHA, PFK-BETA, and PHT2) and carbohydrate metabolism (FBP, PEPC16, SPS1, and SS) in the roots and shoots of Jimian169 and DES926 under low and normal P conditions

The transcripts levels of PAP and PFK were greater under low P in the roots and shoots of both cotton genotypes, however, the increase was high in Jimian169 as compared to DES926 (Fig. 7), indicating a greater P metabolism in Jimian169 under low P. In contrast, low P inhibits the transcript levels of FBP, PEPC, SPS, SS, and PHT in the roots and shoots of Jimian169 and DES926. Irrespective of the P conditions, the transcript levels of genes related to carbohydrate metabolism were high in Jimian169 as compared to DES926, suggesting that Jimian169 can perform better and tolerate low P. The response patterns of FBP, PEPC, SPS, SS, and PHT greatly differed in the plant tissues, where the response was greater in the shoot than root (Fig. 7), indicating more carbohydrate production in the shoot and its subsequent partitioning to the roots for the development of a better root system. However, the response patterns of PAP and PFK were greater in the roots than in shoots, suggesting the high P metabolism to maintain normal growth under low P condition.

### Multivariate analysis

**Fig. 8:**
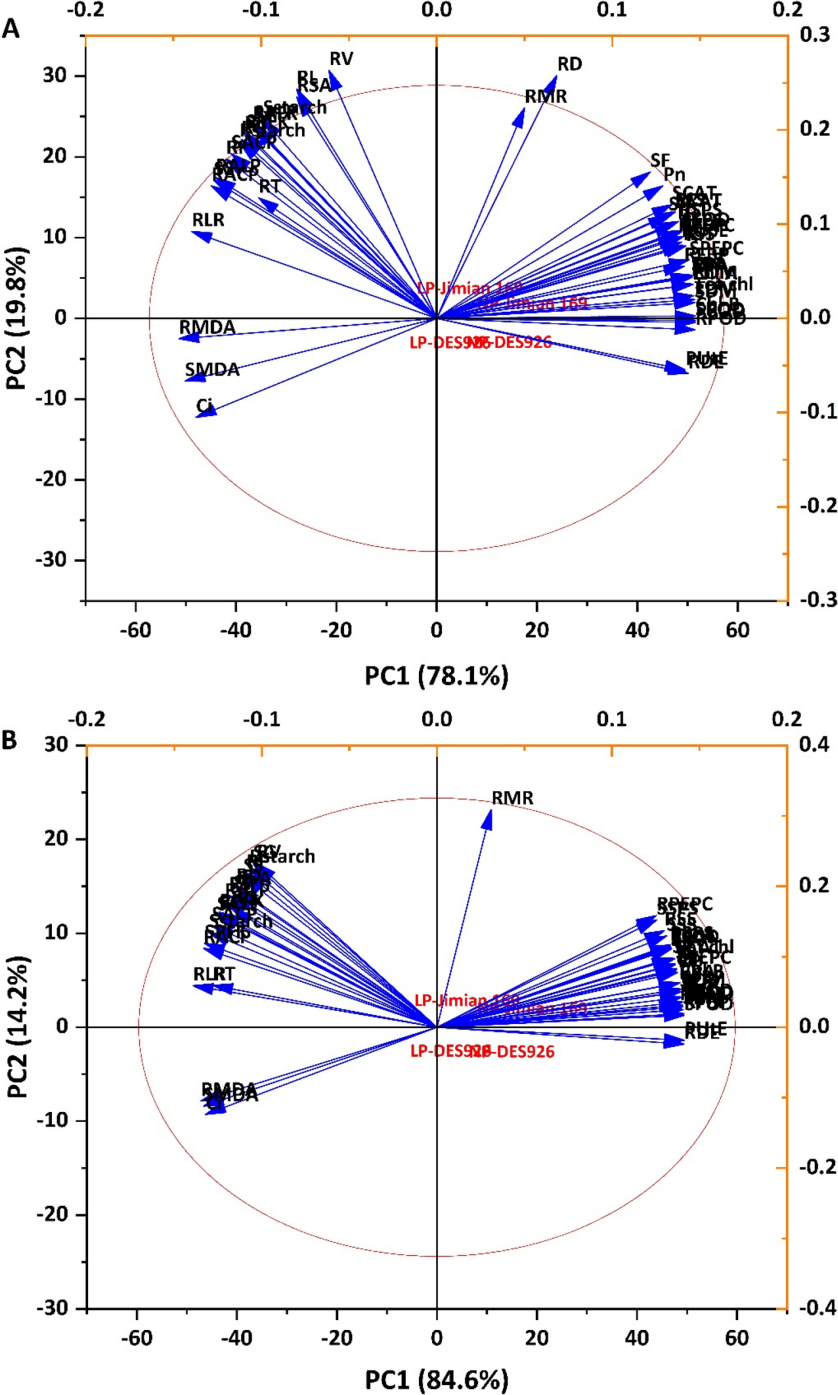
Principal component analysis (PCA) biplot of morphophysiological and biochemical traits of six contrasting low P tolerant cotton genotypes grown under low and normal P conditions in hydroponic (**A**) and pot (**B**) cultures. Shoot length (SL), shoot dry matter (SDM), root dry matter (RDM), total plant dry matter (TDM), root length (RL), root surface area (RSA), root volume (RV), root diameter (RD), root length ratio (RLR), root mass ratio (RMR), root thickness (RT), root density (RDE), chlorophyll a content (Chl a), chlorophyll b contents (Chl b), carotenoid contents (Car), photosynthetic rate (Pn), stomatal conductance (gs), transpiration rate (E), intercellular CO_2_ concentration (Ci), root P concentration (RP), shoot P concentration (SP), total P concentration (TP), root P accumulation (RPA), shoot P accumulation (SPA), total P accumulation (TPA), P uptake efficiency (PUpE), P utilization efficiency (PUtE), root superoxide dismutase activity (RSOD), shoot superoxide dismutase activity (SSOD), root peroxidase activity (RPOD), shoot peroxidase activity (SPOD), root catalase activity (RCAT), shoot catalase activity (SCAT), root malondialdehyde content (RMDA), shoot malondialdehyde content (SMDA), root phosphofructokinase activity (RPFK), shoot phosphofructokinase activity (SPFK), root acid phosphatase activity (RACP), shoot acid phosphatase activity (SACP), root alkali phosphatase activity (RALP), shoot alkali phosphatase activity (SALP), Root glucose content (RG), shoot glucose content (SG), root fructose content (RF), shoot fructose content (SF), root sucrose content (RS), shoot sucrose content (SS), root starch content (Rstarch), shoot starch content (Sstarch), root sucrose synthase activity (RSS), shoot sucrose synthase activity (SSS), root sucrose phosphate synthase activity (RSPS), shoot sucrose phosphate synthase activity (SSPS), root phosphoenolpyruvate carboxylase activity (RPEPC), shoot phosphoenolpyruvate carboxylase activity (SPEPC), root fructose 1,6-bisphosphatase activity (RFBP), and shoot fructose 1,6-bisphosphatase activity (SFBP)

The principal component analysis (PCA) was performed by using the various studied traits to identify the key traits. In hydroponic culture, the PC1 contributed 78.1% and was associated with P conditions, while cotton genotypes were associated with PC2 and shared a variation of 19.8%. Root dry matter, shoot dry matter, total dry matter, chlorophyll a, chlorophyll b, total chlorophyll, carotenoids, transpiration rate, root P accumulation, shoot P accumulation, total P accumulation, root SOD, root POD, shoot POD, and root MDA were the key traits contributing to PC1 and root morphology, enzymes related to P metabolism, and carbohydrate contents mainly contributed to PC2 (Fig. 8A and Table S2). In pot culture, the loading plot of PC1 and PC2 contributed 84.6 and 14.2%, respectively. The traits like shoot length, root dry matter, shoot dry matter, total plant dry matter, single leaf area, root density, chlorophyll b, total chlorophyll, carotenoids, transpiration rate, root P concentration, shoot P concentration, total P concentration, root P accumulation, shoot P accumulation, total P accumulation, P uptake efficiency, P utilization efficiency, root SOD, root POD, shoot POD, root CAT, shoot PEPC, root FBP, and shoot FBP contributed to PC1. However, root length, root surface area, root diameter, root volume, root mass ratio, root PFK, root ALP, shoot ALProot glucose, shoot glucose, root fructose, shoot fructose, root sucrose, shoot sucrose, root starch, and shoot starch contributed to the PC2 (Fig. 8B and Table S2).

**Fig. 9:**
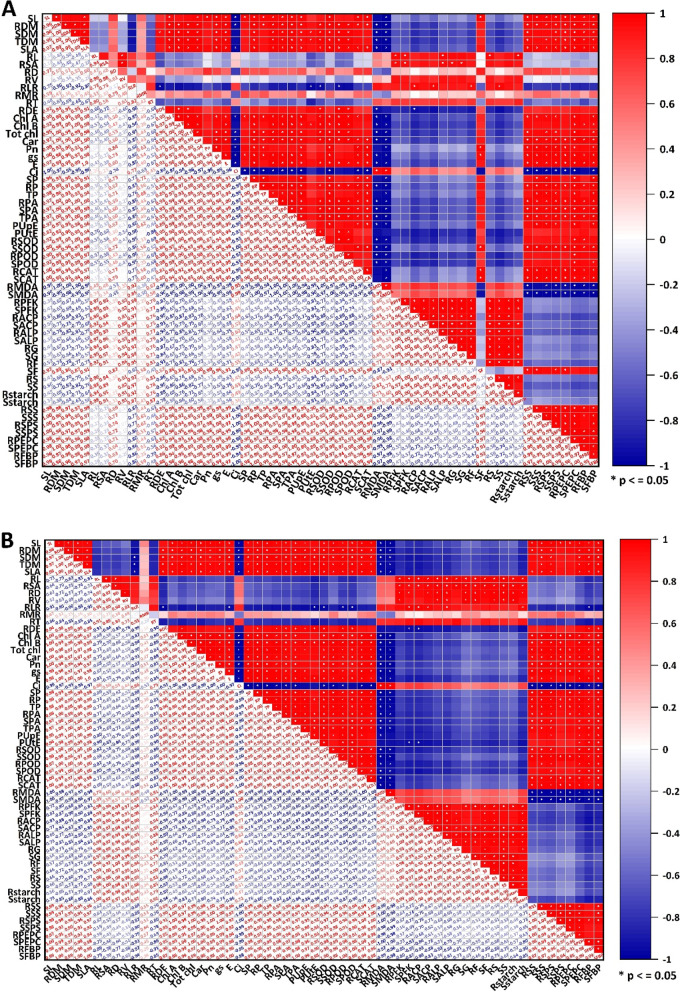
Relationships between morphophysiological and biochemical traits of six contrasting low P tolerant cotton genotypes grown under low and normal P conditions in hydroponic (**A**) and pot (**B**) cultures. Where red color shows a positive correlation and blue color shows a negative correlation. * shows significant differences at *p <* 0.05. All the traits have been defined in fig. 9

In both hydroponic and pot experiments, a strong positive correlation was found among plant morphology, leaf photosynthetic (except intercellular CO_2_ concentration) and chlorophyll traits, PUE traits, and enzymes related to antioxidant system and carbohydrate metabolism. In addition, root morphology (except root diameter), P metabolizing enzymes, and carbohydrate contents were strong positively correlated with each other (Fig. 9). Among the root morphological traits, root length, root volume, and root length ratio had a strong positive correlation with P metabolizing enzymes and carbohydrate contents, suggesting the role of P metabolism and carbohydrates accumulation in the development of a better root system to tolerate low P and thereby increase P uptake (Fig. 9).

## Discussion

Previously, we compared the difference among 385 cotton genotypes in response to low P, and identified the best six contrasting low P tolerant cotton genotypes (data not published). Based on various morphological and physiological indicators, the cotton genotypes Jimian169 and DES926 were identified as strong low P tolerant and weak low P tolerant genotypes, respectively. The current study highlighted that the two contrasting low P tolerant cotton genotypes differed in morphology, physiology, and biochemical adaptation to low P stress. Low P significantly reduced plant morphological traits, however, the reduction was more in DES926 as compared to Jimian169 (Fig. 1). Previous studies have found that low P regulates various plant metabolic processes such as nucleic acid, membrane lipid, protein synthesis, and energy metabolism that ultimately reduced yield [68, 69]. The studies describing the effect of low P on various morphological and physiological traits of cotton genotypes are different in low P tolerance. Low P greatly affects seedlings’ growth and development [4, 60]. The responses of various species and genotypes of the same specie respond differently to low P [70]. In the present study, Jimian169 and DES926 showed differential morphological and physiological responses to low P conditions under both hydroponic and pot cultures.

Roots play an important role in nutrients and water uptake, synthesis of hormones, organic acids, and amino acids that support the growth and yield of the plants [71, 72]. Higher root biomass and root physiology are required for achieving higher production [73] and are important traits that support normal plant growth under stress conditions [74, 75]. Under low P, genotypes partitioned more dry matter into roots [70] which increase root to shoot ratio [76]. Therefore, a lower reduction in shoot dry matter was associated with a better root system in wheat [77]. Because there is a strong interdependence between root and shoot such as dry matter is preferentially allocated to the root for the development of a better root system and in return, a large root system improved P uptake and also remobilize the stored P to shoot [78]. This partitioning of dry matter into roots is important for understanding the efficiency and tolerance of the genotype. As a result, an improved root system is an adaptive strategy under low P condition [79]. In the current study, Jimian169 produced a better root system under low P than DES926 which could efficiently increase the P uptake [80]. Similar results of an increase in root morphological traits were observed in other crops [81, 82] suggesting that improvement in root surface area increases the ability to uptake more P [83]. In contrast, root dry matter was decreased under low P due to a reduction in root diameter and root density and this decrease was more in DES926 as compared to Jimian169 (Fig. 1). Earlier study reported that low P reduced the root diameter of a P-efficient genotype, while increase root surface area for higher P uptake [84]. Therefore, we assumed that Jimian169 can produce a better root system to explore more P under low P condition. In a similar study, it was found that Brassica species increased root surface area to efficiently uptake nutrients from the soil [85].

The current results showed higher carbohydrate contents (glucose, fructose, sucrose, and starch) in the shoots than roots under low P in both hydroponic and pot cultures. However, genotype Jimian169 has comparatively higher carbohydrate contents than DES926 (Fig. 4), which is consistent with the findings in *Phaseolus vulgaris* L. [15]. The increase in carbohydrate contents in the roots of Jimian169 might be attributed to higher translocation from shoot to root [86], higher degradation of sucrose [48], a reduction in hexose phosphorylation [87], and a decrease in the exchange of free cytosolic Pi with triose-phosphate during low P, leading to the accumulation of Calvin cycle intermediates and the flux of starch [88]. It may also be due to the consumption of energy by roots, which is related to active P uptake under low P, and maintenance of the cytoplasmic osmotic potential of the root cells under high P [89]. In contrary with our results, previous study reported that starch concentration in soybean and sugar beet tissues increased with an increase in P concentration [46, 90]. Moreover, high P led to a negative effect on chloroplasts as they were involved in triose P and other phosphorylated metabolites against phosphate. Therefore, high P may reduce the activity of chloroplast and metabolites leading to a reduction in photosynthesis and transitory starch. Despite the accumulation of carbohydrates, the dry matter production of cotton genotypes was significantly lower than that of the normal P (Fig. 1). Reduced dry matter production due to low P has also been identified in rice and maize [91]. These results suggest an alteration of source/sink balance under low and normal P conditions [92]. In summary, low P provided sufficient conditions for carbohydrate accumulation, leading to a decrease in exchangeable Pi in the cytoplasm and increasing the substrate of carbon fixation.

Sucrose is the end product of photosynthesis that is responsible for energy metabolism and the synthesis of complex carbohydrates [93]. It can regulate plant growth under various biotic and abiotic stresses [16]. The homeostasis of carbohydrates particularly sucrose in the different plant organs is regulated by various enzymes that control the synthesis and degradation of carbohydrates according to the plant’s needs. Similarly, it was reported that variation in enzymatic activities involved in sucrose metabolism depends on the activity of sucrose degrading enzymes in the sink as they are responsible for the capacity of the sink organs to import assimilates [94]. Under low P, a similar trend was observed in sucrose biosynthesis (SPS, FBP, PEPC) and degrading (SS) enzymes in the roots and shoots. Among these enzymes, SPS is one of the most important enzymes that regulate sucrose synthesis and sucrose contents in plants [95, 96]. It was further suggested that low P promotes the starch hydrolysis in the shoots and remobilization of carbohydrates to the roots at an early stage, thereby increasing the low P tolerance [95]. It has also been reported that the root morphology of cotton is mainly associated with sink activity and enzymes related to carbohydrate metabolism [97]. For instance, SS is predominant in the accumulation of carbohydrates in the sink and conversion of sucrose to starch [98]. Moreover, it was reported that sucrose degradation in the shoots via SS is associated with a reduction in energy in the invertase pathway to break down sucrose into two molecules of Fru-6-P [99]. Additionally, the involvement of SS in sucrose degradation might be due to its relation with vascular tissues and its presence in the adjacent cells [86]. Subsequently, it can be justified by its role in unloading via the phloem in the developing organs [48]. In case of roots, the phloem unloading was occurring via symplast [100]. Moreover, a large accumulation of starch in the shoot inhibited the sucrose transport and synthesis under low P that reduce plant growth and development [101]. Under low P, the high sucrose loading in the phloem mainly functions to relocate carbohydrates to the roots that increase root size/surface area to efficiently acquire P [17]. Additionally, the transcript levels of genes responsible for these enzymes were also high in both root and shoot of Jimian169 than DES926 (Fig. 7). The increased expression of genes responsible for these enzymes could also be helpful to supply sufficient carbon sources to promote carbohydrate metabolism. Similarly, high expression of genes responsible for glycolysis was observed in the rice roots under low P condition [102]. The expression of PEPC, which feeds carbon skeletons into the TCA cycle, was also increased in white lupin and tobacco under low P [103, 104]. Thus, it may be assumed that an improved root system in Jimian169 under low P is associated with carbohydrate metabolism through various enzymatic activities.

P transporter (PHT) genes were up-regulated and the transcript levels in the root and shoot of Jimian169 were comparatively higher (Fig. 7), indicating that the absorption of P might have increased in Jimian169 under low P condition. Similarly, the transcript level of PAP (ACP) and PFK genes were also increased (Fig. 7), which can support the high release of P from organic P under low P condition. Moreover, the upregulation of these genes indicated that glycolipid and sulfatide might have replaced phospholipid to continue biological function to save P under low P stress [105]. In addition, PAP participates in many metabolic processes at the cellular level like uptake, translocation, and remobilization of P. Similarly, previous studies have reported that low P enhanced PAP activity in green beans [106], lupinus [107], white clover [108], and wheat [109]. In our study, the ACP activity increased in the roots and shoots of both cotton genotypes with a higher increase in Jimian169 than DES926 (Fig. 3). This increase in ACP activity might be due to the hydrolysis of P from phosphate-monoesters, such as nucleic acids and phospholipids, and increased concentration of lipidic, DNA, and RNA fractions [110]. Moreover, the increase in the P metabolizing enzymatic activities in plant roots also increased low P tolerance [111]. In the current study, low P increased the P metabolizing enzymatic activities (PFK, ACP, and ALP) in the roots of both types of cotton genotypes compared to normal P (Fig. 3). In a previous study, a higher ACP activity was noted in the roots of the P-efficient soybean genotype as compared to the P-inefficient genotype under low P [112]. Therefore, we hypothesized that P metabolism is important for increasing low P tolerance.

## Conclusions

**Fig. 10:**
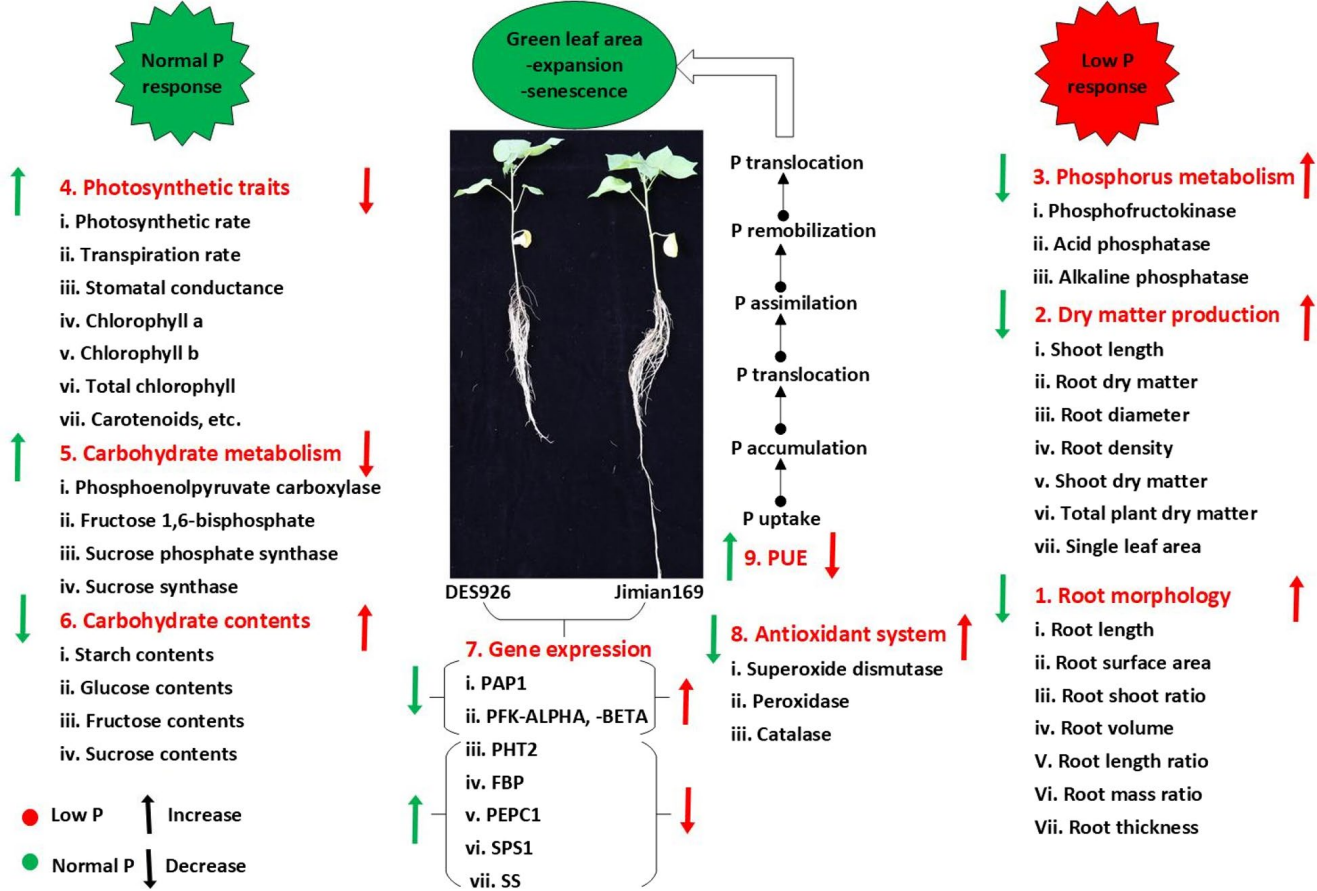
The summary of the physiological mechanism of low P tolerance in cotton. Taken together, a low P tolerant cotton genotype has the characteristics of (1) higher dry matter/carbohydrate accumulation and development of a better root system to efficiently uptake P; (2) higher photosynthetic efficiency and carbohydrate metabolism for biomass production; (3) higher P utilization through P metabolizing enzymes; (4) higher genes expression related to P and carbohydrate metabolism to maintain normal growth; and (5) higher antioxidant enzymatic activities to reduce MDA content and improve low P tolerance

Low P inhibited shoot growth, leaf photosynthesis, and enzymes related to carbohydrate metabolism and antioxidant system. However, low P significantly improved root morphology, P metabolism, and carbohydrate accumulation, particularly in Jimian169. The increase in PUE of Jimian169 might be attributed to its better root system that can uptake P efficiently under low P condition. The multivariate analysis showed that root-related traits, carbohydrate contents, and enzymes related to P metabolism were the most affected by genotype. Further, the variation among the tissue physiological traits was more in roots than in the shoot, suggesting the importance of root over shoot in low P tolerance. Based on these results, the mechanism of low-P tolerance in Jimian169 involved two major stratigies: (1) increasing the ability to use P efficiently by inducing P metabolizing enzymatic activities under low P; and (2) enhancing carbohydrates metabolism and subsequent partitioning into the roots under low P condition (Fig. 10). The transcript level of the key genes responsible for regulating the enzymatic activities in P and carbohydrate metabolism provide useful information and could be used as candidate genes to study the molecular mechanism of low P tolerance in cotton.

## Supplementary Information

**Additional file 1.**

## Data Availability

The datasets supporting the conclusions of this article are included within the article and its additional files.
