# Supplementary material for: Phosphorus and carbohydrate metabolism contributes to low phosphorus tolerance in cotton
Source: BMC Plant Biol. 2023 Feb 16;23:97. doi: 10.1186/s12870-023-04100-6 (PMC9933316; doi:10.1186/s12870-023-04100-6)
Supplement: Supplementary file 1 — Additional file 1. [file 12870_2023_4100_MOESM1_ESM.docx]

Table S1. Gene-specific primers used for RT-qPCR.

| Gene ID | Gene name | Specific primer |
| --- | --- | --- |
| Gh_A12G182500 | PAP1-1-F | CTGCACCTATTAAGATTAAC |
| Gh_A12G182500 | PAP1-1-R | CTGAAGCTTCAAGGACCCT |
| Gh_A13G088000 | PAP1-2-F | TCAACTATTGCTGCTCAA |
| Gh_A13G088000 | PAP1-2-R | CACTGTCACCCAATCTGTATT |
| Gh_A02G109000 | PFK-ALPHA-F | TGATTTGAAATTGGATGGCC |
| Gh_A02G109000 | PFK-ALPHA-R | ACCACCTTTGTAGGACATT |
| Gh_D06G181100 | PFK-BETA-F | CGGAGTAGACAATATTGGATC |
| Gh_D06G181100 | PFK-BETA-R | ATCCCAGCAGGACCACCCTT |
| Gh_A11G377000 | FBP1-1-F | ATGGCATTAACAACAGTATC |
| Gh_A11G377000 | FBP1-1-R | CGCCTGTTCCTGCTTCAACA |
| Gh_D02G079700 | FBP1-2-F | GACGGACCTACGGCTAAGTA |
| Gh_D02G079700 | FBP1-2-R | TCCGTAGAGTAACGTGCGA |
| Gh_A01G054400 | PEPC16-1-F | GCAAGAAGTCATGATTGGGT |
| Gh_A01G054400 | PEPC16-1-R | CCTTAGCAACATTGATAAGC |
| Gh_D01G050600 | PEPC16-2-F | TGAGCTACTTCCATGAAACG |
| Gh_D01G050600 | PEPC16-2-R | CCATCACGATCACCACCCAT |
| Gh_A05G028400 | SS-1-F | ACTACAGCGACGGCAATATC |
| Gh_A05G028400 | SS-1-R | CAATAGATATCTGAATCTGG |
| Gh_A08G136800 | SS-2-F | GCGTCACACAGTGTACCAT |
| Gh_A08G136800 | SS-2-R | GGCTATTAAGTCGGCAGTAA |
| Gh_D06G112600 | SPS1-1-F | CATGCGACGGATAGAGGCTG |
| Gh_D06G112600 | SPS1-1-R | CCAGTACTGGATCAAAGCCA |
| Gh_D13G231900 | SPS1-2-F | ATTTCGTGGAGGAGGTCATT |
| Gh_D13G231900 | SPS1-2-R | CAATCTAGTGTTTCTCTCTTG |
| Gh_A03G158400 | PHT2-1-F | TCCATTAAATAAGAGCTTTC |
| Gh_A03G158400 | PHT2-1-R | AGTCTGAGTAGTACTCTCTTG |
| Gh_D02G177100 | PHT2-2-F | CTTTAGGTCAAGGTGTTGGC |
| Gh_D02G177100 | PHT2-2-R | CATTAGCCACATCATTGGCT |
| AT5G09810 | Gh H 3.3-F | CCTTGTGGGTCTTTTTGAA |
| AT5G09810 | Gh H 3.3-R | AACTGGATGTCCTTGGGC |

Table S2. Principal component analysis (PCA) biplot of various studied traits of contrasting low P tolerant cotton genotypes grown under low and normal P conditions in hydroponic and pot cultures.

| Traits | Hydroponic | | Pot | |
| --- | --- | --- | --- | --- |
|  | PC1 | PC2 | PC1 | PC2 |
| SL | 0.136 | 0.113 | 0.136 | 0.090 |
| RDM | 0.146 | 0.036 | 0.140 | 0.051 |
| SDM | 0.147 | 0.017 | 0.141 | 0.025 |
| TDM | 0.147 | 0.020 | 0.141 | 0.029 |
| SLA | 0.145 | 0.045 | 0.140 | 0.043 |
| RL | -0.080 | 0.244 | -0.125 | 0.160 |
| RSA | -0.080 | 0.235 | -0.115 | 0.196 |
| RD | 0.068 | 0.258 | -0.115 | 0.199 |
| RV | -0.061 | 0.263 | -0.103 | 0.233 |
| RLR | -0.140 | 0.092 | -0.139 | 0.059 |
| RMR | 0.050 | 0.224 | 0.031 | 0.309 |
| RT | -0.102 | 0.128 | -0.128 | 0.059 |
| RDE | 0.143 | -0.058 | 0.141 | -0.024 |
| Chl A | 0.145 | 0.036 | 0.133 | 0.116 |
| Chl B | 0.147 | 0.003 | 0.139 | 0.063 |
| Tot chl | 0.146 | 0.024 | 0.136 | 0.098 |
| Car | 0.145 | 0.045 | 0.137 | 0.081 |
| Pn | 0.129 | 0.141 | 0.134 | 0.114 |
| gs | 0.139 | 0.094 | 0.134 | 0.113 |
| E | 0.147 | 0.020 | 0.141 | 0.029 |
| Ci | -0.137 | -0.105 | -0.132 | -0.124 |
| SP | 0.132 | 0.109 | 0.134 | 0.098 |
| RP | 0.142 | 0.077 | 0.138 | 0.078 |
| TP | 0.136 | 0.100 | 0.136 | 0.089 |
| RPA | 0.146 | 0.044 | 0.138 | 0.062 |
| SPA | 0.145 | 0.046 | 0.139 | 0.050 |
| TPA | 0.145 | 0.046 | 0.139 | 0.051 |
| PUpE | 0.139 | 0.081 | 0.139 | 0.032 |
| PUtE | 0.142 | -0.055 | 0.141 | -0.018 |
| RSOD | 0.147 | -0.004 | 0.141 | 0.036 |
| SSOD | 0.140 | 0.093 | 0.134 | 0.113 |
| RPOD | 0.147 | -0.012 | 0.140 | 0.032 |
| SPOD | 0.147 | -0.002 | 0.141 | 0.017 |
| RCAT | 0.136 | 0.113 | 0.136 | 0.097 |
| SCAT | 0.134 | 0.121 | 0.134 | 0.111 |
| RMDA | -0.147 | -0.022 | -0.135 | -0.105 |
| SMDA | -0.143 | -0.067 | -0.133 | -0.112 |
| RPFK | -0.105 | 0.205 | -0.124 | 0.163 |
| SPFK | -0.109 | 0.197 | -0.133 | 0.119 |
| RACP | -0.128 | 0.141 | -0.133 | 0.112 |
| SACP | -0.117 | 0.175 | -0.129 | 0.145 |
| RALP | -0.126 | 0.150 | -0.121 | 0.178 |
| SALP | -0.104 | 0.207 | -0.126 | 0.159 |
| RG | -0.111 | 0.190 | -0.117 | 0.168 |
| SG | -0.104 | 0.203 | -0.105 | 0.232 |
| RF | -0.120 | 0.169 | -0.109 | 0.219 |
| SF | 0.122 | 0.155 | -0.116 | 0.189 |
| RS | -0.110 | 0.195 | -0.119 | 0.187 |
| SS | -0.127 | 0.147 | -0.111 | 0.214 |
| Rstarch | -0.113 | 0.188 | -0.107 | 0.226 |
| Sstarch | -0.099 | 0.212 | -0.130 | 0.134 |
| RSS | 0.140 | 0.078 | 0.130 | 0.137 |
| SSS | 0.136 | 0.113 | 0.131 | 0.130 |
| RSPS | 0.138 | 0.103 | 0.133 | 0.117 |
| SSPS | 0.139 | 0.092 | 0.126 | 0.153 |
| RPEPC | 0.138 | 0.087 | 0.125 | 0.159 |
| SPEPC | 0.144 | 0.062 | 0.137 | 0.083 |
| RFBP | 0.141 | 0.055 | 0.141 | 0.019 |
| SFBP | 0.140 | 0.087 | 0.140 | 0.054 |
| Eigenvalue | 46.08 | 11.7 | 49.9 | 8.35 |
| Percentage of variance (%) | 78.11 | 19.83 | 84.58 | 14.16 |
| Cumulative (%) | 78.11 | 97.93 | 84.58 | 98.73 |
